# The crosstalk among autophagy, apoptosis, and pyroptosis in cardiovascular disease

**DOI:** 10.3389/fcvm.2022.997469

**Published:** 2022-10-28

**Authors:** Lin Cong, Yunpeng Bai, Zhigang Guo

**Affiliations:** ^1^Academy of Medical Engineering and Translational Medicine, Tianjin University, Tianjin, China; ^2^Department of Cardiac Surgery, Chest Hospital, Tianjin University, Tianjin, China; ^3^Clinical School of Thoracic, Tianjin Medical University, Tianjin, China

**Keywords:** autophagy, apoptosis, pyroptosis, cardiovascular disease (CVD), crosstalk

## Abstract

In recent years, the mechanism of cell death has become a hotspot in research on the pathogenesis and treatment of cardiovascular disease (CVD). Different cell death modes, including autophagy, apoptosis, and pyroptosis, are mosaic with each other and collaboratively regulate the process of CVD. This review summarizes the interaction and crosstalk of key pathways or proteins which play a critical role in the entire process of CVD and explores the specific mechanisms. Furthermore, this paper assesses the interrelationships among these three cell deaths and reviews how they regulate the pathogenesis of CVD. By understanding how these three cell death modes go together we can learn about the pathogenesis of CVD, which will enable us to identify new targets for preventing, controlling, and treating CVD. It will not only reduce mortality but also improve the quality of life.

## Introduction

Cardiovascular disease (CVD) is the leading cause of death in the world, with 18.6 million deaths in 2019, about 31% of all deaths (1). The current reports show that at present, there are 523 million patients with CVD in the world. However, the incidence of CVD is likely to increase substantially due to population growth and aging (1). CVD has a tremendous impact on people’s quality of life, so reducing the burden of CVD is a huge challenge for us. The mechanism of CVD is widely believed to be closely related to cell death and has become a research hotspot in recent years. Different cell death models once were thought to be independent of each other but with the rapid development of molecular biology, their interrelationships have attracted many researchers’ attention. Current evidence suggests that the potential mechanism of cell death is extremely complex, and several modes of cell death interact in the pathogenesis of CVD (2). But now most studies have focused on just one kind of cell death mode and there are few comprehensive and detailed studies describing the interrelationships of different cell death models. This review summarizes the communication and crosstalk among different cell death models and provides a basis for developing more effective methods to prevent and treat CVD in the future.

In the past 3 decades, five types of programmed cell death (PCD) have been discovered: autophagy, apoptosis, pyroptosis, necrosis, and ferroptosis (3). Here we focus on autophagy, apoptosis, and pyroptosis, as these three cell death modes are associated and interactive at multiple levels in CVD. Autophagy and apoptosis have been extensively studied by scientists for many years, and they are now focusing more on a better understanding of pyroptosis in recent years. Table 1 describes the basic cellular morphological characteristics, classification, and associated CVD of the three cell death modes. It has been established that autophagy is an evolutionarily conserved cellular degradation mechanism. Some intracellular components in autophagy, such as misfolded/aggregated proteins and damaged organelles, are encapsulated by autophagosomes and then delivered to lysosomes or vacuoles for degradation (4). Current evidence suggests that most CVDs are related to the activation or inhibition of autophagy (5–11). Apoptosis is an activity and PCD mediated by gene regulation, which can lead to morphological and biochemical changes, including cell shrinkage, plasma membrane blebbing, chromosome pyknosis, nuclear fragmentation, and DNA banding, and ultimately phagocytose cells by phagosomes (12). A large amount of evidence has confirmed that proper regulation of apoptosis has a profound impact on the process of various CVD (13–24). Pyroptosis is an inflammatory type of programmed cell necrosis, in which cell membrane pores are formed, cells rapidly swell and rupture, and pro-inflammatory factors and cell contents are released (25). New evidence suggests that pyroptosis is an important trigger and endogenous regulator of cardiovascular inflammation that may play an important role in the pathogenesis of CVD (26–30). Although these cell death modes are distinct, there is significant crosstalk among them. Therefore, understanding the interrelationships among different cell death models is critical for developing more effective cardiovascular therapeutics.

**TABLE 1:** Cell death pathway and related morphological characteristics and classification and related CVD.

Cell death modes	Basic cellular morphological characteristics	Classification	Related CVD
Autophagy	1. Autophagosome formation 2. Autophagosome and lysosome fusion 3. Autophagosome cleavage	1. Macroautophagy 2. Microautophagy 3. Chaperone-mediated autophagy	Atherosclerosis (AS) (5) Ischemia/reperfusion (I/R) (6, 7) Cardiomyopathy (8) Heart failure (HF) (9) Hypertension and diabetes (10, 11)
Apoptosis	1. Cells round 2. Chromatin condensation 3. Cytoplasmic shrinkage	1. Programmed cell death 2. Natural regeneration of cells 3. Clearance of pathogen-infected cells	AS (13) Post-PCI restenosis (14) I/R (15) Myocardial Infarction (MI) (16) Myocarditis (17) Cardiomyopathy (18) HF (19, 20) Arrhythmia (21) Diabetic Cardiomyopathy (DCM) (22–24)
Pyroptosis	1. Swelling of cells 2. Formation of a large number of vesicles 3. Cell membrane rupture 4. Intracellular content release		AS (26) Hyperlipidemia (27) Hypertension (28, 29) I/R (30)

## Relationship between autophagy and apoptosis

**FIGURE 1:**
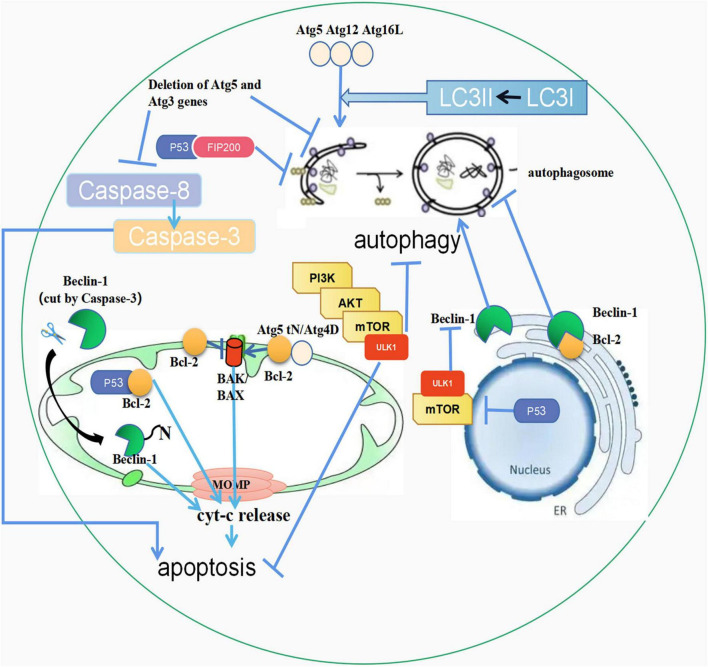
The crosstalk between autophagy and apoptosis. (1) Atg5, Atg12, and Atg16L combine to form a complex, activating LC3-I to LC3-II. Then LC3-II fuses with the autophagic outer membrane to promote the development of autophagy. In addition, the unconjugated Atg5 is cleaved by calpain, and its product, Atg5tN, naturally enters the mitochondria. Subsequently, in the mitochondria, Atg5tN mediates the release of Cyt-c by interacting with BCL-2 proteins, thereby promoting apoptosis. Full-length Atg4D promotes apoptosis, and ΔN63 Atg4D gains GABARAP-L1 coupling autophagy with apoptosis; (2) The PI3K/AKT/mTOR pathway can inhibit both autophagy and apoptosis; (3) Beclin-1 in the endoplasmic reticulum can promote the occurrence of autophagy, but when combined with Bcl-2, it can inhibit autophagy. The cleaved Beclin-1 by Caspase-3 can be translocated to mitochondria, triggering apoptosis; (4) p53 in mitochondria can combine with Bcl-2 to promote apoptosis. p53 in the nucleus promotes autophagy through the mTOR pathway, and p53 in the cytoplasm inhibits autophagy by interacting with FIP200. (5) Deletion of ATG5 and ATG3 inhibits autophagy and apoptosis, and caspase-3 cleaves Beclin-1 to promote apoptosis. ↑ induce; ⟂ inhibit.

Autophagy and apoptosis are conserved intracellular processes that can regulate cell survival and death under stress conditions. In recent years, much emphasis has been placed on dual regulatory proteins linking autophagy to apoptosis (31). Although the interactions of different autophagy-related proteins and apoptosis-related proteins have been found, the underlying regulatory mechanisms have not been clearly understood. Apoptosis and autophagy are double-edged swords and play a dual role in the development of CVD at all stages (Figure 1).

### The crosstalk of autophagy and apoptosis

#### Atg

Current evidence suggests that the anti-thymocyte globulin (Atg) plays an important regulatory role in autophagy and apoptosis (32). During the early stage of autophagosome formation, Atg12-Atg5-Atg16L, a complex formed by covalent bonds, activates microtubule-associated protein 1 light chain 3-I (LC3-I) to microtubule-associated protein 1 light chain 3-II (LC3-II). Subsequently, LC3-II fuses with the outer membrane of the autophagosome, which promotes the expansion of the autophagosome, gradually developing from small vesicle-like and cup-like structures to semi-circular and cyclic structures. However, it’s worth noting that complex formation is not the only way by which Atg proteins promote autophagy. Compared with the above method, other ways are slower during autophagy (33). Atg proteins can accelerate autophagy regulation by forming complexes and promoting apoptosis through the cleavage of products. In apoptotic cells, non-conjugated Atg5 is cleaved by calpain and its product, Atg5tN, enters the mitochondria naturally. Subsequently, Atg5tN mediates the release of cytochrome c (Cyt-c) in the mitochondria by interacting with Bcl-2 proteins (34, 35). In addition, ATG5 gene deletion has been shown to have profound effects on autophagy and apoptosis in mammals. In Atg5-deficient mouse embryonic stem cells, Atg5 proteins lose the ability to interact with Atg12, causing a reduction in the formation of the Atg12-Atg5-Atg16L complex, preventing activation of LC3-1 and ultimately inhibiting the formation of autophagosomes (36). Besides, some studies have corroborated that Atg5-deficient mouse embryonic stem cells are more prone to starvation-induced apoptosis. The autophagy-related gene Atg4 family member (Atg4D) is a substrate of Caspase-3 during apoptosis. Full-length Atg4D is normally inactive, but when overexpressed, it can induce apoptosis through the mitochondrial pathway. After cleavage of Atg4D by Caspase-3, the truncated ΔN63 Atg4D acquires GABARAP-L1 processing activity and becomes hypertoxic, thereby combining autophagy with apoptosis (37, 38).

#### mTOR

The PI3K/AKT/mTOR pathway is a classic signal transduction pathway that promotes cell survival, resists apoptosis, and inhibits autophagy. In the presence of adequate nutrients, signals such as growth factors, glucose, and amino acids can interact with the mTOR complex 1 (mTORC1) signal transduction pathway mediated by serine/threonine phosphorylation, leading to negative regulation of autophagy (39). In this signal transduction pathway, cells are protected from apoptosis due to the synergistic effect (40). However, it is generally accepted that autophagy and apoptosis are antagonistic to each other in the mTOR pathway. Knockdown of glutathione peroxidase 3 (GPX3) has been shown to induce reactive oxygen species (ROS) accumulation and mTOR inhibition, ultimately leading to apoptosis and cardiomyocyte injury. At the same time, since mTOR is inhibited, autophagy is activated to protect cells from damage. Therefore, apoptosis and autophagy are antagonistic to each other in Gpx3-knockdown cardiomyocytes (41).

#### Beclin-1

Beclin-1 is an indispensable regulatory protein with multiple biological functions in the process of autophagosome formation. Non-conjugated Beclin-1 exerts a positive influence on autophagy but an inhibitory effect when bound to Bcl-2 (42). Beclin-1 is a new substrate of Caspase proteins that can be cleaved by Caspase-3, Caspase-6, Caspase-7, Caspase-8, Caspase-9, and Caspase-10, and loses the ability to induce autophagy. Then, the C-terminal fragment of the cleaved Beclin-1 is translocated and penetrates the mitochondria, ultimately promoting Cyt-c release and initiating apoptosis instead. Hence, Beclin-1 plays a dual important role in apoptosis and autophagy (42, 43).

#### P53

P53 is an apoptosis regulator which induces apoptosis through two mechanisms. First, as a transcription factor, p53 can induce and inhibit pro-apoptotic target genes and anti-apoptotic target genes respectively. Moreover, p53 can be translocated to the mitochondria and interact with Bcl-2 family members to induce mitochondrial membrane permeability and Cyt-c release (44). P53 usually exists in the cytoplasm and can be transferred to the nucleus after DNA damage (45). Current evidence suggests that nuclear p53 is an autophagy-promoting factor that induces autophagy by regulating the mTOR pathway in a transcriptional-dependent manner (46). However, in the cytoplasm, p53 inhibits the induction of autophagy. p53 inhibits autophagy by interacting with FIP200 in the cytoplasm, thus preventing the activation of ulk1-fip200-Atg13-Atg101 complex and inhibiting the formation of autophagosome (47).

#### Caspase

Atg3 is involved in autophagosome formation and has a Caspase-8 target in its sequence (48). Mutations in the caspase-8 cleavage site on Atg3 inhibit Caspase-8 cleavage *in vitro* (49). Some studies have suggested that the deletion of Atg5 or Atg3 inhibits the formation of autophagosomes, thereby significantly inhibiting the activation of Caspase-8 and apoptosis (50). Similar to Caspase-8, Caspase-3 can also cleave autophagy proteins. Two Caspase-3 cleavage sites have been found in Beclin-1, which may be involved in the regulation of autophagy and apoptosis. Following Caspase-3 cleavage, the exposed BH3 domain of Beclin-1 may enhance apoptosis by directly binding other anti-apoptotic members in the Bcl-2 family (51).

### The role of autophagy and apoptosis in cardiovascular disease

#### Atherosclerosis

The pathological process of atherosclerosis (AS) includes endothelial cell damage, lipid deposition, foam cell formation, intimal fibrosis, plaque formation, unstable plaque rupture, or erosion (52). Therefore, it is crucial to look for factors that accelerate plaque progression and instability. Some studies have suggested that ruptured plaques are characterized by a bulky and lipid-rich core, a thin fibrous cap, massive cell death with concomitant angiogenesis, and adventitial inflammation (52). Autophagy and apoptosis play non-negligible roles among them. In macrophage-derived foam cells, inhibition of autophagy promotes Caspase-dependent apoptosis *via* activation of Bax, which can reduce the expression of tumor necrosis factor α (TNF-α), interleukin 1β (IL-1β) and IL-6 during AS (53). In cardiovascular endothelial cells (VEC), palmitate-treated mitochondria trigger autophagy and inhibit excessive apoptosis to protect cardiovascular cells (54). Comparative studies demonstrated that when the autophagy of endothelial progenitor cells is inhibited, increased cell viability and decreased levels of apoptosis are observed in coronary heart disease, indicating that the regulation of autophagy on VEC may be related to its maturity. The above results substantiate that moderate activation of autophagy is beneficial to AS in most cases, and the anti-AS effect mediated by autophagy is mainly due to the lipid regulation of macrophages and VECs. At present, available anti-AS drugs can target apoptosis and autophagy, which provides a promising therapeutic direction for AS (55–57).

#### Myocardial infarction

Myocardial infarction (MI) is a type of myocardial necrosis caused by acute and persistent ischemia and hypoxia of the coronary artery (58). MI can damage various structures and functions of the heart, leading to heart failure, arrhythmias, cardiac rupture, and even death. Apoptosis and autophagy make a difference in the occurrence and development of MI, even they are closely related to myocardial injury and ventricular remodeling caused by MI (59). Some studies have suggested that the pro-apoptotic proteins Bax and Caspase-3 are reduced in neonatal rat cardiomyocytes induced by hypoxia-reoxygenation and play an active anti-apoptotic role in promoting autophagy. Inhibition of autophagy weakens the protective effect of autophagy on the heart (60). Therefore, cardiomyocytes can survive under stress by compensatory mechanisms that increase autophagy and inhibit apoptosis. The above process can effectively inhibit MI.

#### Hypertension

Long-term hypertension leads to cardiac systolic dysfunction and a series of pathological events such as increased left ventricular pressure, hypertrophy of cardiomyocytes, abnormal accumulation of extracellular matrix, and formation of myocardial fibrosis. Therefore, the early and reasonable intervention of MI is a focal issue that cannot be ignored in the treatment of hypertension (61, 62). In the process of hypertensive MI, autophagy reduces DNA damage and prevents apoptosis by digesting intracellular impaired components. Autophagy also provides the energy required for apoptosis during the initiation of apoptotic bodies. When the heart lacks the autophagy gene Atg5, it will be hypertrophic, the left ventricular will be systolic and diastolic dysfunction will appear. At the same time, Atg5 is cleaved by calpain to regulate autophagy to form pro-apoptotic fragments (63).

#### Heart failure

Heart failure (HF) is a clinical syndrome in which the heart cannot supply enough blood to meet the body’s metabolic needs. It is considered to be the ultimate common pathway of all heart diseases and a major cause of death in patients with heart disease (64). Some studies have suggested that myocardial cell death plays an important role in the pathogenesis and development of HF. In the process of HF, the increase of autophagy inhibits apoptosis. The mechanism is that autophagy protein Beclin-1 interacts with anti-apoptotic protein Bcl-2 and other pathways, and the effect on myocardial cell survival rate is higher than on apoptosis (65, 66). During the development of HF, apoptosis and autophagy can antagonize each other and exist simultaneously or sequentially with other types of cell death (20, 65, 66).

#### Diabetic cardiomyopathy

Hyperglycemia plays a key role in the pathogenesis and pathological changes of diabetic cardiomyopathy (DCM). Hyperglycemia can directly cause pathological changes in normal cardiomyocytes, leading to cell degeneration, hypertrophy, fibrosis, focal necrosis, and can also increase PCD by affecting mitochondrial function (67). Cardiomyocyte apoptosis can be reduced by adjusting the level of autophagy in DCM. Anti-apoptotic proteins Bcl-2 and Bcl-xL can bind to Beclin-1, which participates in the formation of autophagosomes and regulates cell autophagy and apoptosis (68). In addition, the knockdown of the autophagy gene Atg5 leads to cardiomyocyte apoptosis and increased DCM (69). In the mouse model of DCM, curcumin can interfere with the binding of Beclin-1, Bcl-2, and BIM through the AMPK pathway to activate autophagy and inhibit cardiomyocyte apoptosis (70). The same conclusion can be drawn from other disease models (71, 72). In addition, some studies have suggested that proapoptotic kinase in mouse hearts can inhibit cardiac autophagy (73). The above research results suggest that autophagy is interrelated with apoptosis in DCM.

#### Vascular calcification

Vascular calcification (VC) is a common pathological manifestation of AS, hypertension, diabetic vascular disease, vascular injury, chronic kidney disease, and aging (74). Apoptosis is closely related to calcification. Calcified vascular smooth muscle cells (VSMCs) are prone to apoptosis, which in turn promotes VC (75, 76). In addition, autophagy is also involved in the calcification of VSMCs through the expression of Beclin-1 gene (77). Some researchers have found that the autophagy gene Beclin-1 interacts with the anti-apoptotic gene Bcl-2 during VC, but the functional significance of this interaction remains unclear (78).

#### Drug-induced cardiotoxicity

Cardiotoxicity is mainly due to a variety of drug-induced side effects, leading to damage to the heart. Anthracyclines induce cardiotoxicity by simultaneously modulating autophagy and apoptosis. Doxorubicin (DOX) upregulates autophagy in neonatal rat ventricular myocytes (NRC) (79). DOX has also been shown to promote autophagy by increasing the phosphorylation of adenosine monophosphate-activated protein kinase (AMPK) and inhibiting mTOR (80). However, Dox is also thought to induce cardiotoxicity by inhibiting autophagy. For example, DOX has been found to cause cardiotoxicity by inhibiting autophagy through reduced AMPK phosphorylation and ULK1 dephosphorylation (81). In addition, anthracyclines have been found to damage the myocardium by regulating apoptosis. DOX has been shown to induce p53 overactivation to induce apoptosis by up-regulating apoptotic proteins such as Bax, CytC, and Caspase-3, and down-regulating Bcl-2 expression (82). On the other hand, there are experiments showing that the accumulation of ROS can activate extrinsic apoptotic pathways (83).

## Relationship between autophagy and pyroptosis

**FIGURE 2:**
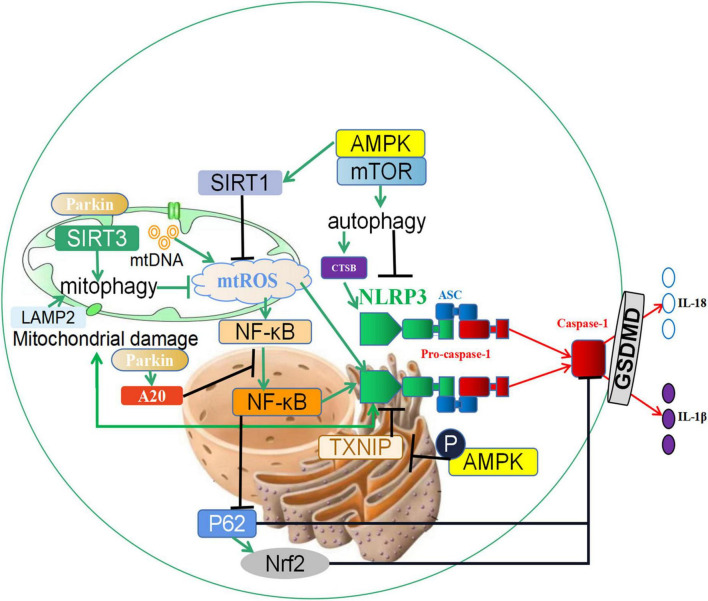
The crosstalk between autophagy and pyroptosis. (1) SIRT3 induces the increase of mitophagy, inhibits the generation of reactive oxygen species (ROS) in mitochondria, and prevents the activation of inflammasome NLRP3. In addition, the expression changes of NLRP3 can reversely regulate mitochondrial damage and ROS generation; (2) Autophagy promotes the increase of CTSB, accelerates the activation of the NLRP3 inflammasome, and induces the occurrence of pyroptosis; (3) Parkin promotes the occurrence of mitophagy, and it also increases the expression of A20, which inhibits the nuclear entry and activation of NF-κB, and reduces the activity of the NLRP3 inflammasome. In addition, NF-κB can also promote the activation of Caspase-1 through the p62 and Nrf2/ARE axis and accelerate the occurrence of pyroptosis. (4) Phosphorylation of the autophagy-related protein AMPK leads to the degeneration of TXNIP and promotes the activation of the NLRP3 inflammasome. Furthermore, the AMPK/mTOR pathway promotes the expression of SIRT1, downregulates reactive oxygen species, and prevents the activation of the NLRP3 Inflammasome. ↑ induce; ⟂ inhibit.

Although there are significant differences between autophagy and pyroptosis in terms of biochemical metabolic pathways and morphology characteristics, a growing body of data suggests that autophagy is inextricably linked to pyroptosis. Autophagy and pyroptosis do not act independently in cells, so it means that they influence and regulate each other. The balanced regulation of the two mechanisms can maintain the stability of AS plaques, thereby providing a potential solution for the prevention and treatment of CVD (Figure 2).

### The crosstalk of autophagy and pyroptosis

#### Mitophagy-reactive oxygen species regulates pyroptosis

In previous literature, increased mitochondrial reactive oxygen species (mtROS) has been closely related to the activation of the NLR family pyrin domain containing 3 (NLRP3) inflammasome (84, 85). Mitophagy is an autophagic response that specifically targets damaged and potentially cytotoxic mitochondria. When mitophagy is inhibited, damaged mitochondria fail to clear and release mtDNA and mtROS, which can directly activate the NLRP3 inflammasome (86). In turn, the activated NLRP3 inflammasome can lead to mitochondrial membrane rupture and mtROS release, further aggravating mitochondrial damage and inflammation (87). There is a feedback loop between mtROS and NLRP3 inflammasome. Hence, mtROS plays a regulatory role in the activation of the NLRP3 Inflammasome. In addition, recent evidence suggests that new substances are involved in the regulation of mtROS on pyroptosis. Cong et al. showed that sirtuin 3 (SIRT3), a critical mitochondrial deacetylase, could promote mitophagy, inhibit ROS increase, and prevent NLRP3 inflammasome activation to protect lipid-laden macrophages from stress (26). According to Liu’s research, berberine, a nature-derived alkaloid compound, could regularly induce mitophagy and inhibit NLRP3 inflammasome activation (88).

#### Autophagy/CTSB/NLRP3

CTSB is an intracellular cysteine protease that exists mainly in lysosomes and is correlated with the autophagic flux in the cytoplasm. Curcumin induces up-regulation of autophagy and then induced the release of CTSB, the activation of NLRP3 inflammasome and pyroptosis through autophagy. It has been shown that inducing pyroptosis through autophagy/CTSB/NLRP3/Caspase-1/Gasdermin D (GSDMD) signaling pathway can effectively inhibit cell proliferation and migration (89). This finding contradicts other studies and points out the pathway of autophagy promoting pyroptosis for the first time, indicating that autophagy is also a double-edged sword in the regulation of pyroptosis.

#### Parkin, NF-κB, and p62

In recent years, the mechanism by which the PINK1-Parkin pathway initiates mitophagy has been revealed (90–92). With the participation of parkin, damaged mitochondria are modified by ubiquitination, further promoting the expression of parkin in the cytoplasm (93). In addition to the above regulatory mechanism, parkin is involved in inhibiting the activation of inflammasomes, whereby parkin increases the expression of anti-apoptotic signaling protein 20 (A20), and then inhibits nuclear entry and activation of nuclear factor kappa-B (NF-κB). Finally, NLRP3 inflammasome activity was decreased (94). Furthermore, the NF-κB-p62 mitophagy pathway inhibits Caspase-1 and attenuates pyroptosis, leading to reciprocal regulation of autophagy and pyroptosis (86). Further studies have indicated that Nrf2, a nuclear factor activated by p62, is involved in macrophage pyroptosis (86). The overactivation or inhibition of Nrf2/ARE signaling exacerbates or alleviates pyroptosis, whereby p62 levels are regulated by Nrf2 feedback. Blockade of autophagy promotes pyroptosis of ox-LDL-treated macrophages *via* the p62/Nrf2/ARE axis, providing a new therapeutic target for AS (95).

#### Autophagy-related protein

The regulation of energy metabolic balance is mediated by several related signaling pathways in which the AMP-activated protein kinase (AMPK)/mTOR signaling pathway constitutes a switch in anabolic and catabolic processes. The AMPK/mTOR signaling pathway is an important regulatory pathway for autophagy. It has been reported that Metformin can promote autophagy *via* the above pathway to inhibit the activation of NLRP3 inflammatory corpuscle and play a protective role in the heart (96). In an effort to understand the signaling mechanisms underlying the antipyroptotic properties of Exendin-4, researchers found that blockade of AMPK, an oxidative stress sensor, can reduce the antipyroptotic property of Exendin-4. Phosphorylation of AMPK leads to the degeneration of TXNIP, thereby promoting the activation of the NLRP3 inflammasome (97). Colchicine plays a crucial role in alleviating the intracellular inflammatory response and NLRP3 inflammation activation, attenuating cellular oxidative stress and levels of pyroptosis in endothelial cells *via* regulating AMPK/sirtuin 1 (SIRT1) signaling, which may be a concrete mechanism for the secondary prevention of CVD (98).

### The role of autophagy and pyroptosis in cardiovascular disease

#### Atherosclerosis

AS is a chronic inflammatory disease associated with lipid metabolism disorders (99). Pyroptosis is an important component of inflammatory reaction, which is closely related to the occurrence and development of AS. Therefore, inhibiting pyroptosis by regulating autophagy has become a new target for the treatment of AS (100). Ample evidence suggests that enhanced mitophagy can prevent pyroptosis by elevating SIRT3 to promote autophagy and inhibit ROS production, which may be a viable strategy to combat pyroptosis-mediated inflammation in AS (26). Studies related to melatonin have shown that melatonin (MT) has anti-inflammatory properties. Treatment of high-fat diet-induced ApoE^–/–^ mice with MT indicates a reduction in both NLRP3 activation and IL-1β secretion in AS. However, 3-methyladenine, an autophagy inhibitor, could reverse this protective process. Accordingly, MT inhibits AS progression by enhancing autophagy and anti-inflammatory properties. In addition, MT has been shown to reduce NLRP3 inflammasome expression by eliminating ROS in macrophages, thereby reducing AS plaques and increasing stability (101). When cells are starved, autophagy can activate Caspase-1 and inflammasome through an Atg5-dependent pathway, increase the release of inflammatory factors, and aggravate inflammatory tissue damage. This phenomenon has only been documented in yeast, and the exact relationship between pyroptosis and autophagy in mammals and their specific roles in different stages of AS warrants further study (102).

#### Myocardial ischemic/reperfusion injury

Myocardial ischemia/reperfusion injury (MI/RI) is defined as the additional myocardial injury caused by reperfusion after the ischemic myocardium is reperfused. MI/RI is mainly involved in multiple mechanisms such as oxidative stress, intracellular calcium overload, and inflammatory response, leading to cardiomyocytes eventually reaching the obstructed coronary arteries with PCD (103). It has been demonstrated that the regulatory role of autophagy-related proteins Beclin-1 regulates Caspase-4 activation and pyroptosis. At the same time, Beclin-1 overexpression also reduces the level of the inflammatory factor IL-1β and it can promote autophagy by regulating the expression of p62 and LC3II, thereby protecting human cardiac microvascular endothelial cells (HCMECs) from damage (104). In addition, a study showed that Simiaoyong An Decoction (SMYAD) had a protective effect on myocardial ischemia/reperfusion injury by activating autophagy and inhibiting pyroptosis, and improving the cardiac function of cardiomyocytes. The mechanism is that SMYAD increases the protein expression rate of LC3B-II/LC3B-I in H/R cardiomyocytes, reduces the protein expression rate of p-mTOR/mTOR, and downregulates the expression of Caspase-1, NLRP3, and IL-1β (105).

#### Vascular calcification

In recent years, a large number of studies have found that VC is an active, multifactorial, and biological regulation of physiological proceses (106). Many studies have confirmed the role of autophagy in the process of vascular calcification. *In vivo* studies have shown that Irisin can induce autophagy and restore autophagic flux in calcified VSMCs. The addition of autophagy inhibitors attenuates the inhibitory effect of Irisin on β-GP-induced ROS production, NLRP3 inflammasome activation, pyroptosis, and calcification in VSMCs. *In vivo* studies have shown that Irisin treatment promotes autophagy, downregulates ROS level, and thereby inhibits pyroptosis and medial calcification. This finding suggests that Irisin can protect against VC by inducing autophagy and inhibiting pyroptosis (107, 108).

#### Diabetic cardiomyopathy

Diabetes can cause a variety of complications, including DCM, and is closely related to the increased morbidity of HF and arrhythmia. DCM is a common complication of diabetes, which can lead to cardiac hypertrophy and subsequent HF. Chronic inflammation of the diabetic heart leads to the loss of cardiomyocytes and subsequent cardiac injury. Metformin, an antidiabetic drug widely used in the treatment of type 2 diabetes, can exert cardioprotective effects through multiple pathways. The experimental results showed that metformin treats diabetes by reducing the expression of mTOR, NLRP3, Caspase-1, IL-1β, and GSDMD. But *in vivo* and *in vitro* administration of autophagy protein AMPK inhibitors can reverse the therapeutic effect. Mechanistically, the results demonstrate that metformin can activate AMPK, thus improving autophagy *via* inhibiting the mTOR pathway and alleviating pyroptosis in DCM (96).

## Mutual regulation of apoptosis and pyroptosis

**FIGURE 3:**
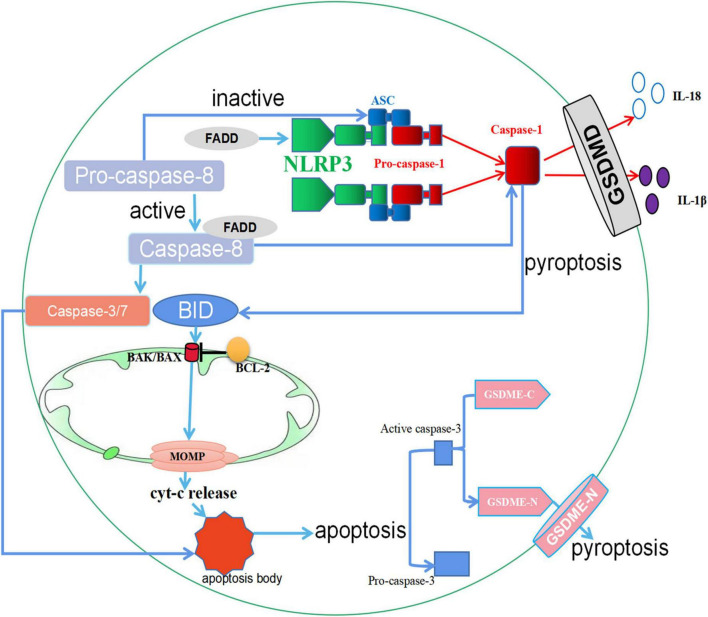
The crosstalk between apoptosis and pyroptosis. (1) When GSDMD is expressed, Caspase-1 protein is activated, and the activated Caspase-1 protein cleaves GSDMD and eventually leads to pyroptosis. However, in the absence of GSDMD in macrophages, activated Caspase-1 protein can cleave BID, leading to the activation of MOMP, the release of Cyt-c, and downstream apoptotic signaling. In cells where GSDME is present, apoptosis promotes Caspase-3 cleavage of GSDME, ultimately leading to pyroptosis. (2) FADD activates both NLRP3 and Caspase-1 proteins to promote pyroptosis. In addition, the secretion of FADD can activate Caspase-8. Activated Caspase-8 promotes apoptosis through Caspase-3/7, and inactive Caspase-8 upregulates pyroptosis by triggering ASC specks. ↑ induce; ⟂ inhibit.

Apoptosis and pyroptosis are PCD exhibiting chromatin condensation, nuclear condensation, and Caspase-dependence. Nonetheless, cells undergoing pyroptosis exhibit cell swelling, cell membrane blistering, and other cell lysis phenomena, like necrosis. Up to now, few studies have explored the relationship between apoptosis and pyroptosis (Figure 3).

### The crosstalk of apoptosis and pyroptosis

#### Caspase-GSDM

Apoptosis is a genetically regulated PCD, and apoptotic cells that are not scavenged progress to pyroptosis. Some basic studies have revealed the relevant mechanisms by which this occurs. Caspase-1-GSDMD is a classical pathway regulating apoptosis and pyroptosis. In the presence of GSDMD in cells, Caspase-1 proteins are activated by macromolecular signaling complexes that bring inactive Pro-caspase-1 proteins together and promote their proximity-induced autoactivation and proteolytic processing. Activated Caspase-1 proteins can cleave a pore-forming protein, GSDMD, which promotes the release of inflammatory mediators such as IL-18 and IL-1β, ultimately leading to pyroptosis (109). However, when GSDMD is absent in macrophages, the activated Caspase-1 proteins can cleave BID, a member of Bcl-2 family proteins, leading to the activation of MOMP, the release of Cyt-c, and downstream apoptotic signaling (109). In addition, Caspase-3-Gasdermin E (GSDME) is similar to Caspase-1-GSDME in function. The existing studies have shown that during apoptosis, activated Caspase-3 proteins cleave GSDME into a GSDME-C fragment and a GSDME-N fragment, where the GSDME-N fragment mediates progression to pyroptosis (110).

#### Caspase-8 and Fas-associated death domain

The Fas-associated death domain (FADD) is not only a central part of diverse death signaling pathways but is also involved in various physiological and pathological processes (111). Caspase-8 is the molecular switch for apoptosis and pyroptosis. Activated caspase-8 promotes apoptosis, while inactive caspase-8 accelerates pyroptosis by triggering the formation of ASC specks (112). FADD activates NLRP3 and Caspase-1 proteins during the inflammation process to promote pyroptosis. The secretion of FADD can activate Caspase-8, and the activated Caspase-8 will activate Caspase-3 and Caspase-7, increasing apoptosis to achieve the mutual regulation of cell pyroptosis and apoptosis (113). Similarly, experiments have also demonstrated that FADD regulates this process of apoptosis and pyroptosis. RIPK1 in association with FADD and Caspase-8 triggers nucleotide-binding oligomerization NLRP3 inflammasome-dependent and Caspase-8-mediated cleavage of GSDMD and the execution of pyroptosis. This complex also engages Caspase-8-mediated apoptosis, and inhibition of Caspase-8 activity promotes RIPK3-mixed lineage kinase domain-like pseudokinase (MLKL)-dependent necroptosis (114).

### The role of pyroptosis and apoptosis in cardiovascular disease

#### Myocardial infarction

Myocardial infarction is a high-mortality disease involving structural, electrical, systolic changes in cardiac tissue, and is often associated with PCD (115). During MI, PCD occurs *via* apoptosis, pyroptosis, and other pathways (116). Apoptosis is the best-characterized PCD process, which involves chromatin condensation, DNA fragmentation, cell membrane blistering, cell shrinkage, and formation of apoptotic bodies (117). It is interesting to note that increased NLRP3 in primary newborn rat cardiac fibroblasts (RCFs) has been shown to recruit more ASC proteins, promote Caspase-1 hydrolysis, and mediate the release of GSDMD proteins, eventually causing pyroptosis. The expression of BAX/Bcl-2 ratio, Caspase-3, and GSDMD proteins is increased. GSK-3 β-mediated activation of the NLRP3 inflammasome/Caspase-1/IL-1β pathway leads to cardiomyocyte and primary human cardiac fibroblasts (CF) apoptosis and pyroptosis to promote the development of MI (118).

#### Myocardium injury

MI is not only closely related to apoptosis. During myocardial ischemia-reperfusion, the pyroptosis of myocardial fibroblasts, vascular endothelial cells, and macrophages will lead to the release of intracellular inflammatory factors and metabolites to the extracellular matrix, aggravating MI (119). Li has established that apigenin can protect H9c2 cells from myocardial injury induced by ischemia-hypoxia (I/H). The protective effects are most likely related to the reduction of pyroptosis, apoptosis, and pro-inflammatory cytokines (120). This study had some limitations. The specific mechanism of apigenin on the pyroptosis and apoptosis of I/H-induced myocardial injury in H9c2 cells is still not clear (120).

## Summary and discussion

Building on this comprehensive understanding of the molecular mechanisms of autophagy, apoptosis, and pyroptosis, it is critical to put these into the context of tissue homeostasis and pathology in CVD. While remaining significantly different, there is coexistence and mutual crosstalk among these three cell deaths. Indeed, autophagy, apoptosis, and pyroptosis constitute a pluralistic, coordinated cell death system in which one pathway can flexibly compensate for the other (121). It is very significant to study the molecular mechanisms of the three cell death modes and find the common points of the relationship and regulation among the three modes for researching the pathogenesis and prevention of CVD.

In the process of CVD, apoptosis and autophagy play a dual role, that is, cardiovascular protective effect and disease progression promotion. Furthermore, the effect, which is caused by the above cell deaths, makes a significant difference in various CVDs, even in diverse stages of the same CVD. The molecular mosaic between autophagy and apoptosis is relatively intricate and the related pathogenesis of CVD is relatively complex (57). In most cases, autophagy is a protective form of autophagy, and it is mostly inhibitory when it comes to apoptosis. But sometimes autophagy also induces apoptosis. On the contrary, apoptosis normally protects the cardiovascular system by clearing out senescent cells, and it can both promote and inhibit autophagy (122). However, both excessive autophagy and excessive apoptosis play a role in damaging the cardiovascular system. Unlike autophagy and apoptosis, the negative role of pyroptosis in CVD is well-defined. Pyroptosis not only causes local inflammation, but also leads to an amplification of the inflammatory response (123). In recent years, a variety of drugs have been discovered to treat CVD by inhibiting pyroptosis and inflammatory responses (124). It has been described above that autophagy can inhibit the inflammatory response to pyroptosis through multiple pathways in most cases. Although the relationship between apoptosis and pyroptosis has not been thoroughly studied, the existing research results show that regulating the level of apoptosis can indeed affect pyroptosis. Therefore, studying the specific molecular mechanisms by which autophagy and apoptosis regulate pyroptosis may provide important targets for the treatment of inhibiting the inflammatory response caused by pyroptosis in CVD.

Few studies have been conducted on the crosstalk among pyroptosis and the other two cell death modes, emphasizing that further research is needed. Furthermore, the related pathogenesis of CVD is still very obscure, and it only stays in appearance. Apart from CVD, cell death is also involved in other diseases, including respiratory and digestive tumors. However, current studies have only reported cell death in a single type of cell, and the effects and differences among different types of cells are poorly informed (3). Next, we can consider the mutual regulation of the three cell death modes in other diseases, which may provide targets for the treatment of CVD caused by other diseases. At the same time, drug-induced CVD is also a pivotal part of CVD. The process of drug action *in vivo* is very complex. In the past, we usually only considered the impact of drug-induced changes in a certain way of death on the heart, while ignoring the possible occurrence of other death modes. A holistic comprehension of the specific regulatory mechanisms among each death mode provides new targets for preventing, controlling, and treating CVD, which contribute to reducing mortality and improving the quality of life of millions of people around the world.

## Author contributions

ZG contributed to the conception and design, final approval of the manuscript, and obtaining funding. YB contributed to the design, literature search, analysis and interpretation, and obtaining funding. LC performed literature search, collected relevant material, organized and summarized, and wrote the original manuscript. All authors contributed to the article and approved the submitted version.
